# Omadacycline for the treatment of severe pneumonia caused by *Chlamydia psittaci* complicated with acute respiratory distress syndrome during the COVID-19 pandemic

**DOI:** 10.3389/fmed.2023.1207534

**Published:** 2024-01-08

**Authors:** Dao-Xin Wang, Ling-Xi Xiao, Xin-Yu Deng, Wang Deng

**Affiliations:** ^1^Department of Respiratory and Critical Care Medicine, Second Affiliated Hospital of Chongqing Medical University, Chongqing, China; ^2^Chongqing Medical Research Center for Respiratory and Critical Care Medicine, Chongqing, China

**Keywords:** ARDS, *Chlamydia psittaci*, mNGS, omadacycline, severe pneumonia

## Abstract

**Introduction:**

*Chlamydia psittaci* infection in humans is a rare cause that mainly present as community-acquired pneumonia. Severe *Chlamydia psittaci* pneumonia can lead to acute respiratory distress syndrome (ARDS), septic shock, or multiple organ dysfunction with a mortality rate of 15%–20% before accurate diagnosis and targeted treatment. Metagenomic next-generation sequencing (mNGS) has an advantage in achieving early diagnosis. In the study, omadacycline implementation was described to provide a better understanding of effectiveness in severe psittacosis pneumonia with ARDS.

**Methods:**

Sixteen patients with severe psittacosis pneumonia with ARDS were selected between September 2021 and October 2022. They were diagnosed using mNGS and treated with omadacycline. Retrospective analysis of clinical manifestations, laboratory data, disease progression, diagnostic tool, treatment, and prognosis was summarized.

**Results:**

Common symptoms included fever, dyspnea, and cough. All patients developed ARDS, accompanied by septic shock (43.7%) and pulmonary embolism (43.7%). Laboratory data showed normal leucocytes, increased creatine kinase isoenzyme, and decreased albumin with liver dysfunction in most patients. All patients had increased neutrophils, C-reactive protein, procalcitonin, and D-dimer with decreased lymphocytes. Airspace consolidation, ground glass opacity, and pleural effusion were found on chest CT. mNGS results were obtained in 24–48 h to identify the diagnosis of Chlamydia psittacosis. All patients received mechanical ventilation with omadacycline treatment. Fourteen patients experienced complete recovery, while the other two patients died from multidrug-resistant bacterial infection and renal failure.

**Conclusion:**

mNGS has a significant value in the diagnosis of *Chlamydia psittaci* infection. Timely treatment of omadacycline can improve prognosis and provide a promising new option for the treatment of severe *Chlamydia psittaci* pneumonia with ARDS.

## Introduction

*Chlamydia psittaci* is an intracellular parasitic pathogen transmitted from the inhalation of aerosols from contaminated bird substances to humans, which is common in adults and rare in children (1). Psittacosis is an infectious zoonosis caused by *Chlamydia psittaci* from poultry, wild birds, and some other animals (2). *Chlamydia psittaci* pneumonia accounts for less than 5% of community-acquired pneumonia (CAP) in hospitals (3). *Chlamydia psittaci* was considered the common pathogens for severe CAP in China (4). The severity of pneumonia ranges from atypical flu-like symptoms to fatal conditions with acute respiratory distress syndrome (ARDS) and/or multiple organ dysfunction (5–7). Few cases of clinically diagnosed *Chlamydia psittaci* pneumonia with ARDS have been reported or may even be underestimated probably due to atypical clinical presentation, low sensitivity of routine laboratory tests, and low awareness of the disease (8, 9).

The typical manifestation may include high fever, dry cough, headache, myalgia, chills, and gastrointestinal symptoms. In severe conditions, symptoms can progress to respiratory failure, endocarditis, jaundice, and neurological complications. In addition to the symptoms and an exposure history, the diagnosis of *Chlamydia psittaci* requires a laboratory examination meeting any one of the three criteria: (1) isolation of *Chlamydia psittaci* from respiratory secretions; (2) IgM antibody against *Chlamydia psittaci* titer of 1:16 or higher by micro-immunofluorescence (MIF) detection; (3) a 4-fold or greater increase in antibody titer between serum samples collected 2 weeks apart, using a complement fixation test or MIF (10). Specific diagnostic tests such as polymerase chain reaction (PCR) and pathogenic culture are only available in some specialized microbiology laboratories. Therefore, the limitation of diagnosis raises a great concern (11, 12).

Metagenomic next-generation sequencing (mNGS) has been widely used to detect different types of potential microorganisms including viral, bacterial, fungal, or parasitic pathogens (13). The application of mNGS technology provides a helpful method for the diagnosis of infectious diseases using a culture-independent approach.

Most importantly, mNGS has the ability to diagnose atypical pathogens within 48–36 h that usually takes a long time in traditional culture methodologies, which leads to a favorable clinical outcome based on early diagnosis and more effective antibiotic therapy (14). For critically ill patients, mNGS has recently been highlighted as the most promising tool for the accurate diagnosis of rare pathogenic infections, particularly for severe pneumonia. Recent studies have shown that the mNGS provides high specificity and sensitivity for species-level identification with a less level in false-positive and false-negative results compared with traditional methods (14, 15).

Omadacycline is a novel tetracycline for the treatment of CAP approved by the United States Food and Drug Administration in 2018 (16). There is still a lack of clinical data on omadacycline treatment for severe *Chlamydia psittaci* pneumonia with ARDS. In the study, severe CAP caused by *Chlamydia psittaci* with ARDS was summarized for the clinical characteristics, laboratory data, treatments, and outcomes. Furthermore, we evaluate the contribution of omadacycline in the treatment of severe psittacosis pneumonia.

## Patients and methods

### Study design

We conducted a retrospective case review of 16 patients with severe CAP caused by *Chlamydia psittaci* admitted to the Second Affiliated Hospital of Chongqing Medical University between September 2021 and October 2022 when COVID-19 pandemic was supposed to spread in China. All cases were diagnosed with ARDS based on clinical manifestations, laboratory data, and mNGS. For each case, clinical characteristics, laboratory examination, imaging, diagnosis, treatment, outcomes, and follow-up data were recorded from the electronic medical system. The study was approved by the Ethics Committee of the Second Affiliated Hospital of Chongqing Medical University. All data were anonymized prior to analysis.

### Diagnostic criteria for severe psittacosis pneumonia with ARDS

The diagnosis of severe psittacosis pneumonia with ARDS should be considered as the following criteria: (1) meet the criteria for severe CAP (17); (2) identify specific fragment DNA of *Chlamydia psittaci* by mNGS; (3) fulfill the Berlin definition of ARDS (18); (4) have negative results for other causative organisms by routine pathogen tests with bronchoalveolar lavage fluid (BALF), sputum, and blood.

The criteria for severe CAP include one major criterion or more than three minor criteria. Major criteria contain septic shock with the need for vasopressors and respiratory failure requiring mechanical ventilation. Minor criteria contain respiratory rate ≥30 breaths/min, PaO_2_/FiO_2_ ratio ≤250, multilobar infiltrates, confusion/disorientation, uremia (blood urea nitrogen level ≥20 mg/dL), leukopenia (white blood cell count <4,000 cells/μL), thrombocytopenia (platelet count <100,000/μL), hypothermia (core temperature <36°C), and hypotension requiring aggressive fluid resuscitation.

The severity of ARDS is based on the degree of hypoxemia: mild (200 mm Hg <PaO_2_/FIO_2_ ≤300 mm Hg), moderate (100 mm Hg <PaO_2_/FIO_2_ ≤200 mm Hg), and severe (PaO_2_/FIO_2_ ≤100 mm Hg).

### mNGS detection method

1. Sample processing and DNA extraction: clinical samples (1.5–3 mL BALF or 3–4 mL blood) were collected according to standard procedures. Samples were treated with enzymes at 4°C for liquefaction within 24 to 48 h. Then the samples were transferred to new microcentrifuge tubes and broken by vortex mixer with glass beads on a horizontal platform vigorously agitated at 1,600 × g for 30 min. DNA was extracted from a 0.3 mL sample using the TIANamp Micro DNA Kit (Tiangen Biotech, China). The extracted DNA samples were used for the construction of DNA libraries.
2. Library preparation and sequencing: an Agilent 2100 Bioanalyzer was used as quality control of DNA libraries to analyze the length of the inserted fragments. The construction of DNA libraries were performed using transposase-mediated methods (Vision Medicals, China) and assessed by a Qsep1 biofragment analyzer before sequencing. Qualified DNA libraries were loaded into the sequencing chip and performed on the NextSeq 550Dx sequencing platform (Illumina, San Diego, CA).
3. Data analysis: high-quality data was obtained by removing low-quality reads and short reads (length <40 bp), followed by subtraction of human host sequences mapped to the human reference genome (hg38 and YH sequences) using Burrows–Wheeler Aligner software. The remaining data by eliminating low-complexity reads were compared in the special microbial database. The classification reference data were downloaded and optimized from an open database such as NCBI, China National GenBank Database or EMBL. Data of suspected pathogenic microorganisms with the numbers of coverage rate, strictly mapped reads and depth were then analyzed and produced according to the Microbial Genome Database. The final result is exported and interpreted with microbiology and clinical background. The diagnosis was determined based on the clinical manifestations, imaging, pathogens and other essential laboratory tests.

## Results

### Patients’ characteristics

Sixteen patients with severe *Chlamydia psittaci* pneumonia complicated with ARDS were enrolled. Among them, eleven were men with five women. Their median age was 65 (range 39–76) years. Nine patients (56.2%) had underlying diseases, such as hypertension, diabetes, hepatitis B, or chronic obstructive pulmonary disease (COPD). Six patients had a smoking history. All patients (100%) had a history of exposure or close contact with parrots, pigeons, or poultry. Eight of them raised parrots, pigeons, chickens, or ducks at home for years as a definite exposure history. The rest had a contact history such as live poultry market visits or live poultry for cooking. All patients were diagnosed with *Chlamydia psittaci* detected by mNGS (Table 1). The detection of novel coronavirus disease 2019 (COVID-19) by reverse transcription PCR using nasal swabs in all patients was negative. Other respiratory pathogens for CAP such as influenza virus, respiratory syncytial virus, adenovirus, *Legionella pneumophila*, *Mycoplasma pneumoniae*, and *Chlamydia pneumoniae* were negative by pathogenic detection at admission.

**Table 1:** Basic characteristics of patients with severe psittacosis pneumonia.

Patient No.	Gender	Age (y)	Exposure contact	Underlying disease	Smoking history	Confirmed method	Severity of ARDS
1	Male	75	Raised pigeon	Hypertension	Yes	mNGS	Severe
2	Male	68	Parrot contact	Diabetes, hepatitis B	Yes	mNGS	Moderate
3	Female	67	Raised parrot	Hypertension, diabetes	No	mNGS	Moderate
4	Female	39	Raised parrot	None	No	mNGS	Moderate
5	Male	74	Ducks contact	Hepatitis B	No	mNGS	Mild
6	Male	61	Parrot contact	None	No	mNGS	Mild
7	Female	48	Raised chickens and ducks	None	No	mNGS	Mild
8	Male	69	Parrot contact	COPD	Yes	mNGS	Severe
9	Male	73	Parrot contact	Hypertension	Yes	mNGS	Moderate
10	Female	67	Pigeon contact	None	No	mNGS	Mild
11	Male	65	Raised parrot	Hypertension	No	mNGS	Severe
12	Male	71	Raised parrot	Hypertension	Yes	mNGS	Severe
13	Female	59	Raised pigeon	None	No	mNGS	Severe
14	Male	70	Parrot contact	None	Yes	mNGS	Moderate
15	Male	58	Raised chickens	Diabetes	No	mNGS	Severe
16	Male	76	Parrot contact	None	No	mNGS	Moderate

COPD, chronic obstructive pulmonary disease; mNGS, metagenomic next generation sequencing.

### Clinical symptoms

Fever (100%), dyspnea (87.5%), and cough (68.7%) were the most common symptoms in patients. All patients had a recurrent fever higher than 39°C. Eleven patients had cough without apparent expectoration. Six patients had chill (37.5%) and headache (37.5%). Seven patients had myalgia (43.7%). Fourteen patients developed respiratory failure due to progressive dyspnea. All patients presented with ARDS (mild 25%, moderate 37.5%, and severe 37.5%) with seven patients developing septic shock (43.7%) during hospitalization. The average of acute physiology and chronic health evaluation and sequential organ failure assessment scores were 17 (range 8–28) and 5.8 (range 2–12), respectively (Tables 1, 2). The physical examination of the patients was non-specific including increased respiratory rate, enhanced tactile vocal fremitus, and moist rales on the affected lung.

**Table 2:** Clinical symptoms of patients with severe psittacosis pneumonia.

Patient No.	Fever	Cough	Chill	Headache	Myalgia	Dyspnea	Septic shock	P/F ratio (mm Hg)	APACHE II	SOFA
1	Yes	Yes	No	No	No	Yes	Yes	97	28	10
2	Yes	Yes	No	No	No	Yes	Yes	105	18	8
3	Yes	No	No	Yes	Yes	Yes	No	130	13	3
4	Yes	Yes	Yes	Yes	No	Yes	Yes	110	16	6
5	Yes	No	No	No	Yes	No	No	250	8	4
6	Yes	Yes	No	No	No	No	No	243	9	3
7	Yes	Yes	Yes	No	Yes	Yes	No	224	11	2
8	Yes	Yes	Yes	No	Yes	Yes	Yes	99	24	12
9	Yes	No	No	Yes	No	Yes	No	190	18	5
10	Yes	No	No	Yes	No	Yes	Yes	216	24	8
11	Yes	Yes	Yes	Yes	No	Yes	No	87	20	5
12	Yes	Yes	Yes	No	No	Yes	No	99	17	3
13	Yes	Yes	No	No	Yes	Yes	Yes	75	18	10
14	Yes	Yes	No	Yes	No	Yes	No	185	13	2
15	Yes	No	No	No	Yes	Yes	No	84	24	4
16	Yes	Yes	Yes	No	Yes	Yes	Yes	148	14	8

APACHE II; acute physiology, age and chronic health evaluation; P/F, PaO_2_/FiO_2_, SOFA; sequential organ failure assessment.

### Laboratory examinations

The average of white blood cell (WBC) count in all patients was 8.70 × 10^9^/L. Three patients presented an elevated WBC count (18.7%), whereas thirteen patients presented a normal WBC count on admission. All patients had increased neutrophils, procalcitonin (PCT), and C-reactive protein (CRP) with a decreased percentage of lymphocytes. Patients had a mean neutrophil percentage of 84.3%, an average PCT level of 3.42 ng/mL, and an average lymphocyte percentage of 6.15%, respectively. Fourteen patients (87.5%) had an increase in creatine kinase isoenzyme (CK-MB) with the highest level of 14,722 U/L. Most patients had hepatic dysfunction, and among them, eleven patients (68.7%) had increased alanine aminotransferase (ALT) and fifteen patients (93.7%) had increased aspartate aminotransferase (AST). A decreased albumin was presented in fifteen patients (93.7%) with an average level of 29.1 g/L. All patients had an increased level of plasma D-dimer. In addition, one patient had an elevated level of creatinine (Table 3). Seven patients (43.7%) were diagnosed with pulmonary embolism (PE) by pulmonary angiography.

**Table 3:** Laboratory testing of patients with severe psittacosis pneumonia.

Patient No.	WBC (4–10 × 10^9^/L)	N% (45–75%)	CRP (<10 mg/L)	PCT (0.02–0.05 ng/mL)	L% (20–50%)	CK-MB (0–25 U/L)	Albumin (35–50 g/L)	ALT (7–40 U/L)	AST (13–35 U/L)	Cre (40–130, μmol/L)	D-dimer (0–550, ng/mL)	PE	Chest CT
1	7.53	89.2	136.2	6.56	4.7	196	35.7	53	52	56.5	1548.3	No	Consolidation
2	25.98	95.3	155.73	8.56	2.9	125	27.3	19	58	92.8	2723.2	No	Consolidation
3	8.83	91.5	159.77	0.803	5.3	93	32.5	59	42	69.9	3879.3	Yes	Consolidation
4	6.72	85.6	>200	0.16	10.2	87.4	28.4	110	140	51.9	2805.8	No	Consolidation
5	10.14	92.3	141	1.16	3.3	14.6	34.1	33	113	59.9	685.1	No	Lobe lesion
6	11.17	97.1	>200	17.92	1.5	2.8	26.7	152	122	61.6	4086.4	Yes	Consolidation
7	5.64	91.8	>200	0.993	6.4	54	31	114	113	96.8	689	No	Lobe lesion
8	5.62	78.6	155.9	0.93	12	218.9	32.5	23	64	52.2	563.5	No	Consolidation
9	7.85	84	149.75	0.162	8.9	300	26.2	107	131	54.9	8263.9	Yes	Lobe lesion, Pleural effusion
10	6.55	97.1	>200	9.77	1.5	47	26.6	54	77	48.6	2,800	No	Consolidation
11	6.12	84.1	>200	3.7	10.1	14,722	34.7	155	429	77.8	2880.8	No	Consolidation
12	9.09	8.29	>200	0.799	4	321	27.9	32	55	52.2	3335.8	Yes	Consolidation
13	8.69	90.7	>200	0.52	6.5	31.0	19.3	13	32	60	1814	Yes	Consolidation
14	5.68	87.7	186.14	0.08	8.5	57	26.7	58	99	55.2	1990	No	Consolidation
15	4.94	91.6	>200	2.05	7.2	676	25.3	87	118	254.2	2278.1	Yes	Lobe lesion
16	5.29	92.1	127.67	0.627	5.5	1938	31	55	48	74.2	2717.8	Yes	Consolidation

WBC, white blood cell; N, neutrophils; L, lymphocyte; CRP, C-reactive protein; PCT, procalcitonin; CK-MB, creatine kinase isoenzyme; ALT, alanine aminotransferase; AST, aspartate aminotransferase; Cre, creatinine; PE, pulmonary embolism; CT, computed tomography.

### Imaging examination

**Figure 1:**
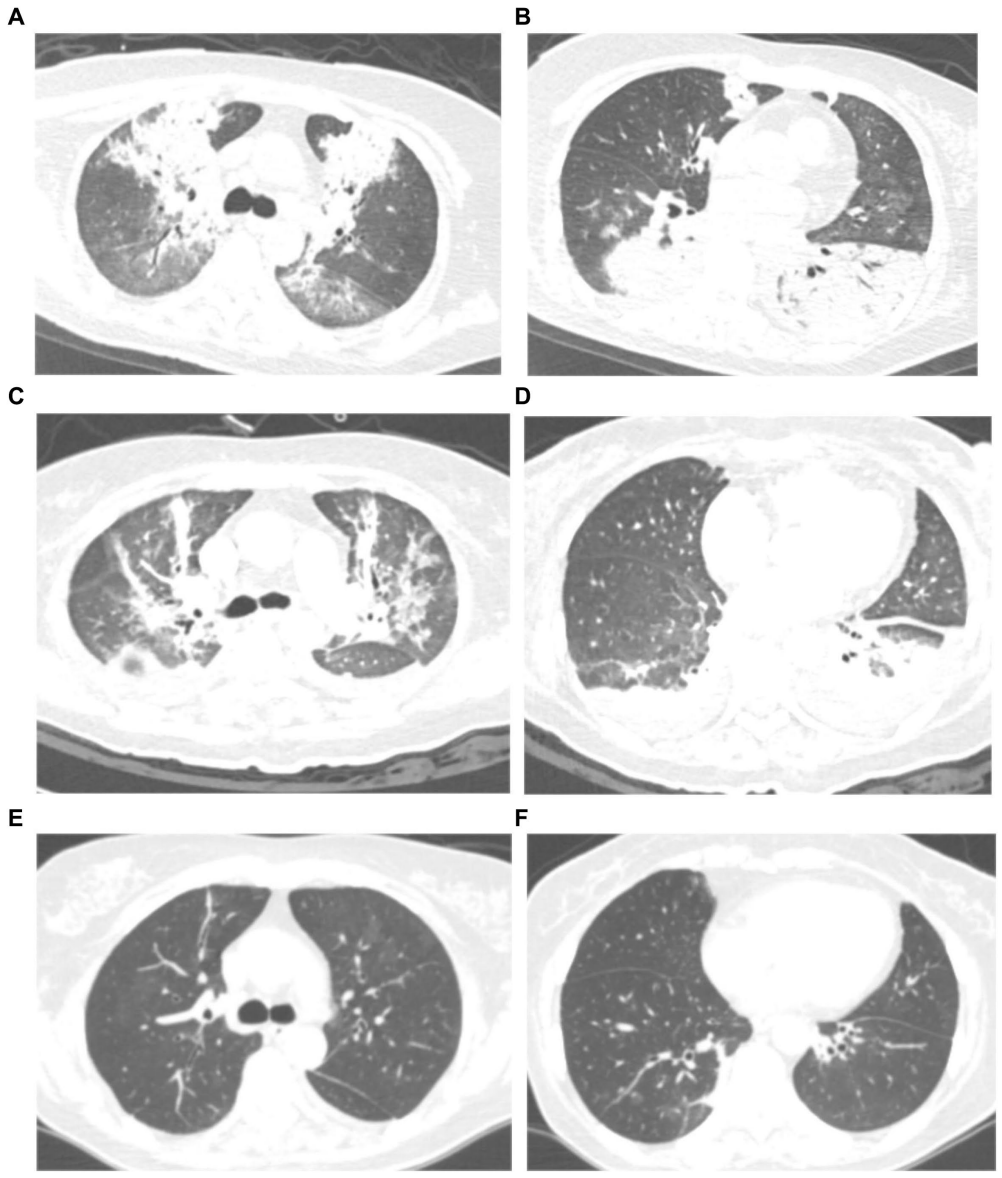
Chest computed tomography (CT) scan of a 59 years-old woman with severe psittacosis pneumonia (No. 13 patient). The initial CT scan (on admission) shows diffused consolidation with air bronchogram of both lungs **(A,B)**. Consolidation gradually decreased after targeted treatment in CT scan (12 days after admission) **(C,D)**. The consolidation completely disappeared on follow-up (28 days after admission) with lung recovery **(E,F)**.

**Figure 2:**
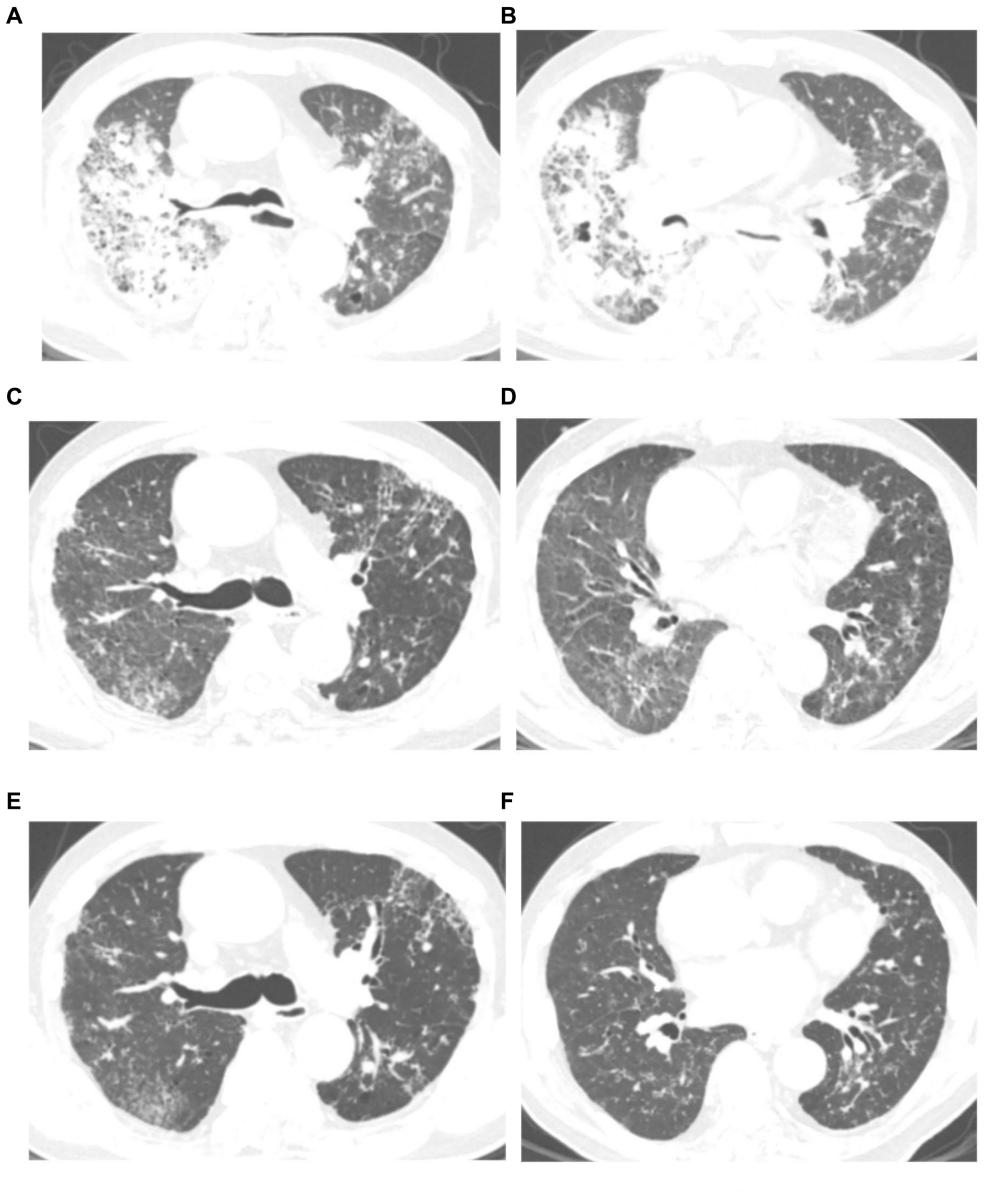
Chest computed tomography (CT) scan of a 76 years-old man with severe psittacosis pneumonia (No. 16 patient). The initial CT scan (on admission) shows diffused consolidation with ground-glass opacity of the right lung **(A,B)**. Consolidation gradually decreased after targeted treatment in CT scan (14 days after admission) **(C,D)**. The consolidation completely disappeared on follow-up (28 days after admission) with lung recovery **(E,F)**.

All patients had inflammatory lesions on the chest computed tomography (CT) examination on admission. Consolidation with air bronchogram or ground-glass opacity (75%) can be observed bilaterally in the lungs of most patients. Three patients had lesions in the upper lobe of the lung without infiltrates in other lobes. Moreover, pleural effusion was also found in one patient. After treatment, the lung lesions gradually disappeared with no residual fibrosis (Figures 1, 2).

### mNGS results

Bronchoalveolar lavage was conducted on all patients by bronchoscopy. Samples of BALF or sputum were collected for mNGS detection. BALF collection can reflect the pathogenic infection of the lungs due to extraction from the bronchi and the low possibility of sample contamination. The results of mNGS showed that *Chlamydia psittaci* identified in all patients with also some other microorganisms at the same time (Table 4). The sensitivity and detection rate of *Chlamydia psittaci* were low because of its intracellular growth and small extracellular release into BALF, sputum, or blood. Pathogenic bacteria such as *Streptococcus oralis*, *Neisseria mucosa*, *Prevotella intermedia*, and *Streptococcus anginosus* should be considered as colonization or contamination due to fewer detection.

**Table 4:** mNGS results of patients with severe psittacosis pneumonia.

Patient No.	Sample type	Data vol (No. of reads)	Detected pathogen(s) (No. of species-specific reads)
1	BALF sputum	24,274,859	*Chlamydia psittaci* (292), *Streptococcus oralis* (2), *Klebsiella pneumoniae* (1)
2	BALF	12,003,511	*Chlamydia psittaci* (115), *Candida albicans* (19)
3	BALF	30,398,231	*Chlamydia psittaci* (373)
4	BALF	28,215,284	*Chlamydia psittaci* (221), *Human alphaherpesvirus* 1 (2)
5	BALF	34,919,252	*Chlamydia psittaci* (69), *Candida albicans* (9)
6	BALF	18,194,616	*Chlamydia psittaci* (28)
7	BALF	20,545,068	*Chlamydia psittaci* (34), *Human alphaherpesvirus* 1 (3)
8	BALF sputum	27,924,632	*Chlamydia psittaci* (1297), *Prevotella intermedia* (23), *Neisseria mucosa* (5)
9	BALF	27,043,045	*Chlamydia psittaci* (3477)
10	BALF	17,988,072	*Chlamydia psittaci* (150), *Human betaherpesvirus* (3)
11	BALF	19,495,853	*Chlamydia psittaci* (72), *Candida albicans* (2), *Staphylococcus epidermidis* (5)
12	BALF	35,447,319	*Chlamydia psittaci* (45)
13	BALF	28,514,379	*Chlamydia psittaci* (26), *Klebsiella pneumoniae* (9)
14	BALF	68,207,678	*Chlamydia psittaci* (11), *Human alphaherpesvirus* 4 (4)
15	BALF	14,842,044	*Chlamydia psittaci* (3650), *Klebsiella pneumoniae* (6), *Haemophilus influenzae* (30), *Human alphaherpesvirus* 4 (5), *Ureaplasma parvum* (4)
16	BALF sputum	39,570,000	*Chlamydia psittaci* (2973), *Streptococcus anginosus* (13)

BALF, bronchoalveolar lavage fluid.

**Figure 3:**
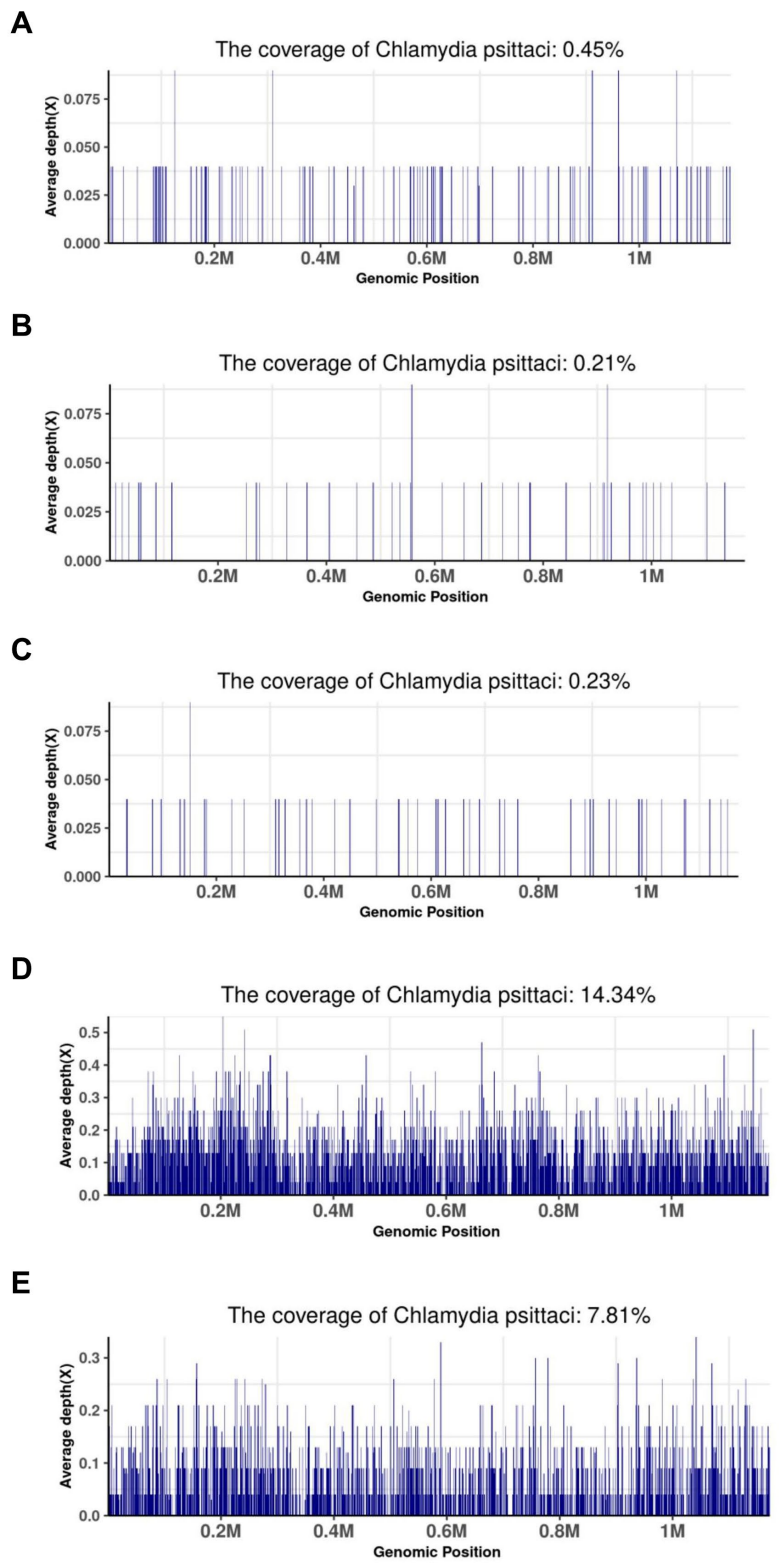
Metagenomic next-generation sequencing results of some patients. **(A)** BALF sample of patient 12; **(B)** BALF sample of patient 13; **(C)** BALF sample of patient 14; **(D)** BALF sample of patient 15; **(E)** BALF and sputum sample of patient 16.

The coverage of mNGS in some patients is shown in Figure 3. Forty-five specific *Chlamydia psittaci* sequences covered 0.45% of the total *Chlamydia psittaci* genome were detected by mNGS in the BALF sample of patient 12. Twenty-six specific *Chlamydia psittaci* sequences covered 0.21% of the total *Chlamydia psittaci* genome were detected by mNGS in the BALF sample of patient 13. Eleven specific *Chlamydia psittaci* sequences covered 0.23% of the total *Chlamydia psittaci* genome were detected by mNGS in the BALF sample of patient 14. A total of 3,650 specific *Chlamydia psittaci* sequences covered 14.34% of the total *Chlamydia psittaci* genome were detected by mNGS in the BALF sample of patient 15. A total of 2,973 specific *Chlamydia psittaci* sequences covered 7.81% of the total *Chlamydia psittaci* genome were detected by mNGS in the BALF and sputum samples of patient 16 (Figure 3).

### Treatment process

**Figure 4:**
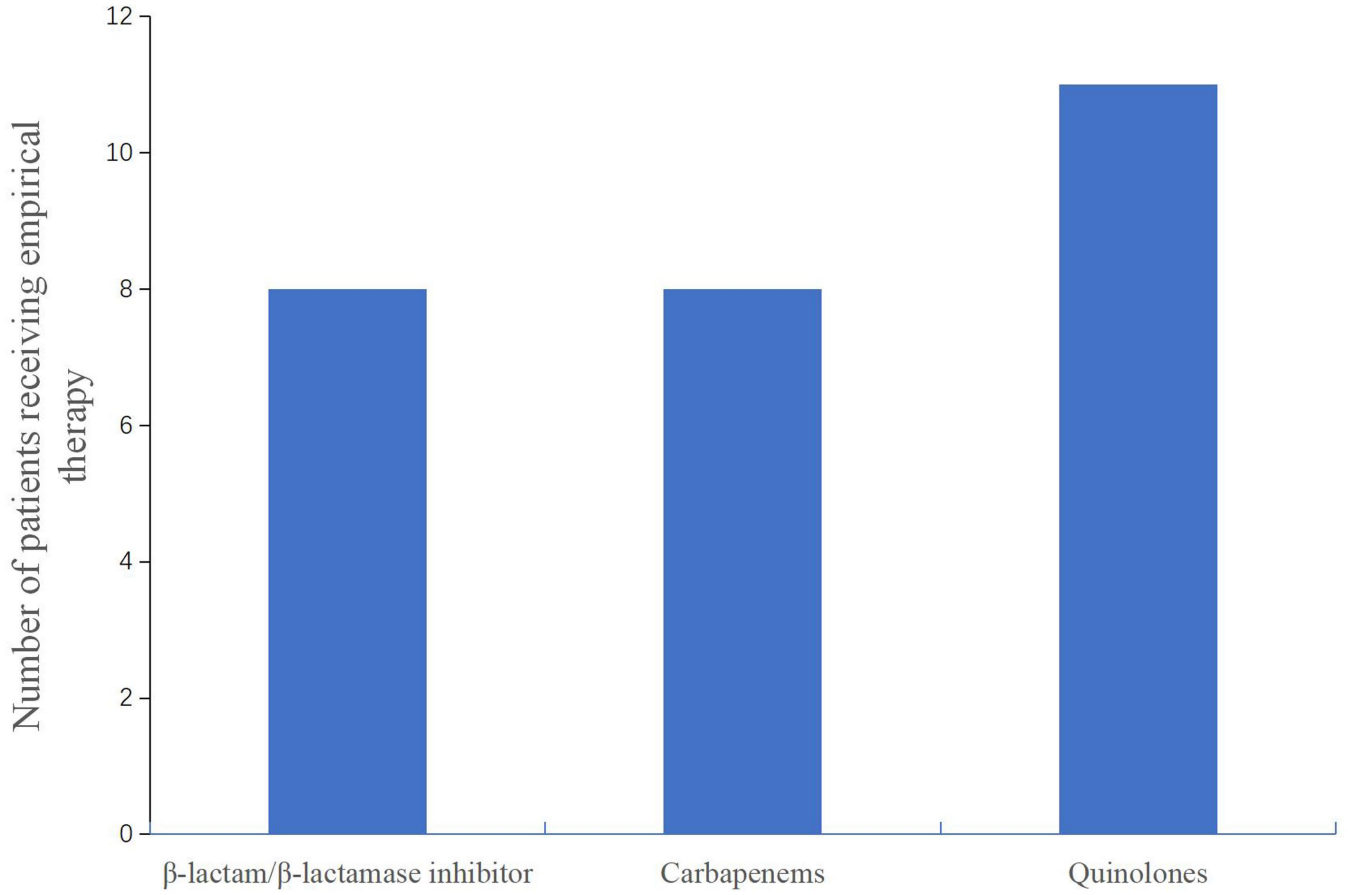
Number of patients receiving empirical antibiotic therapy on admission.

After admission to the hospital, the patients were subjected to bronchoscopy and were collected BALF for mNGS test. Before the results of mNGS, all patients received empirical antibiotic therapy on admission, according to the CAP management guideline (17), including β-lactam/β-lactamase inhibitor and combinations of carbapenems or quinolones (Figure 4). Eight patients were initially receivedβ-lactam/β-lactamase inhibitor or carbapenems, respectively. Eleven patients were initially administered quinolones. The patients did not have sufficient efficacy with the empirical antibiotic therapy due to their clinical conditions not improved or even gradually deteriorated, leading to progression of ARDS. After the confirmation of *Chlamydia psittaci* infection by mNGS, antibiotic therapy was changed to omadacycline for all patients (Table 5). Omadacycline was recommended for the treatment of severe psittacosis pneumonia for at least 2 weeks (7). In addition, some patients not only had psittacosis pneumonia but were also infected with other pathogens through mNGS testing. For these patients, the corresponding antibiotics were applied accordingly (Table 5). It is worth mentioning that patients with severe illness were treated with carbapenems or polymyxin B. Since one patient (No. 1) continued to have multidrug-resistant *Acinetobacter baumannii*, polymyxin B was administered (Table 5).

**Table 5:** Clinical outcomes of patients with severe psittacosis pneumonia after treatment.

Patient No.	WBC (4–10 × 10^9^/L)	N% (45–75%)	CRP (<10 mg/L)	PCT (0.02–0.05 ng/mL)	P/F ratio (mm Hg)	Initial antibiotic treatment	Adjustment of antibiotic	Outcome
1	4.24	74.4	41.87	3.25	84	Cefoperazone/tazobactam	Biapenem + omadacycline + polymyxin B	Death
2	6.32	68.6	2.52	0.049	324	Meropenem + moxifloxacin	Meropenem + omadacycline + caspofungin	Good
3	4.19	59.8	5.34	0.022	308	Piperacillin/tazobactam	Biapenem + omadacycline	Good
4	7.81	72.7	7.61	0.036	312	Cefoperazone/tazobactam	Biapenem + omadacycline	Good
5	6.33	73.8	9.96	0.052	345	Piperacillin/tazobactam	Omadacycline + caspofungin	Good
6	9.8	75	3.15	0.048	323	Meropenem + moxifloxacin	Omadacycline	Good
7	6.79	72.7	7.92	0.035	309	Cefminox + levofloxacin	Omadacycline	Good
8	7.56	73	3.93	5.044	58	Biapenem + moxifloxacin	Biapenem + omadacycline	Death
9	6.01	75	9.55	0.031	318	Piperacillin + moxifloxacin	Piperacillin + omadacycline	Good
10	5.24	65.4	4.58	0.038	363	Biapenem	Piperacillin/tazobactam + omadacycline	Good
11	7.84	74.7	4.13	0.011	306	Biapenem + moxifloxacin	Biapenem + omadacycline + caspofungin	Good
12	4.84	62.7	7.26	0.055	324	Imipenem + moxifloxacin	Imipenem + omadacycline	Good
13	9.04	70.1	5.43	0.049	332	Meropenem + moxifloxacin	Meropenem + omadacycline	Good
14	6.85	67.3	5.83	0.033	339	Piperacillin + levofloxacin	Piperacillin + omadacycline	Good
15	6.86	57.4	5.1	0.011	302	Biapenem + moxifloxacin	Biapenem + omadacycline	Good
16	5.28	72.2	9.32	0.023	341	Cefoperazone + levofloxacin	Cefoperazone + omadacycline	Good

WBC, white blood cell; N, neutrophils, L, lymphocyte; CRP, C-reactive protein; PCT, procalcitonin; P/F, PaO_2_/FiO_2_.

All patients who developed ARDS received mechanical ventilation (MV), including 10 patients with invasive MV and 6 patients with non-invasive MV. Lung-protective ventilation strategy with low tidal volume, high PEEP, sedation, and analgesia was performed. Four patients received high-flow nasal cannula oxygen therapy after the patients’ condition improved with mechanical ventilation. Seven patients (PaO_2_/FIO_2_ ratio <150 mmHg) were performed prone position ventilation with an average of 4.6 days as recommended previously (19). During the treatment, 4 patients received continuous renal replacement therapy (CRRT) because of acute renal injury, of which one patient had diabetes for years.

### Outcomes

After omadacycline treatment, the clinical symptoms including fever, cough, headache, and dyspnea gradually improved. Inflammatory indicators such as WBC, neutrophils percentage, CRP, PCT, and PaO_2_/FiO_2_ ratio were dropped to near normal (Table 5). In addition, CK-MB, ALT, AST, and D-dimer were returned to normal. Two patients (No. 1and 8) were eventually died (Table 5). The cause of death was possibly attributed to the secondary infection of multidrug-resistant *Acinetobacter baumannii* of patient No. 1 and severe infection of *Chlamydia psittaci* with uncontrolled inflammation of patient No. 8, which contributed to the aggravation of septic shock and renal failure requiring continuous renal replacement therapy. In addition, a longer time from the onset to diagnosis of the two patients may lead to delay in treatment with poor outcome compared with other patients. In surviving patients, consolidations on chest CT were absorbed with full recovery of discharge from the hospital.

The treatment duration of all patients is presented in Table 6. The mean time from the onset of the illness to diagnosis was 6.6 days (range 2–13 days). The median time from illness to respiratory failure was 6.6 days (range 3–15 days). The longest duration of MV was 15 days, and the shortest duration of MV was 4 days. The mean time from illness to ICU stay was 7.8 days (range 5–15 days). The longest duration from the illness to hospital stay was 16 days.

**Table 6:** Treatment duration of patients with severe psittacosis pneumonia.

Patient No.	Admission date (year-month-day)	Days from onset to diagnosis	Days from illness to respiratory failure	Mechanical ventilation duration (days)	Days from illness to ICU stay	Days from illness to hospital stay	Prone position duration (days)	Oxygen therapy method	CRRT
1	2022-10-19	13	15	15	15	15	7	MV	Yes
2	2022-01-20	2	3	7	5	14	4	MV	No
3	2022-09-17	7	14	6	7	16	3	MV/HFNC	No
4	2022-03-18	4	6	5	8	12	3	MV	No
5	2022-10-16	7	8	5	5	13	No	MV/HFNC	No
6	2022-09-23	6	5	6	6	12	No	MV	No
7	2022-10-30	6	4	4	7	11	No	MV	No
8	2022-12-21	10	7	12	12	12	6	MV	Yes
9	2022-11-01	4	10	4	7	10	No	MV	No
10	2022-10-06	5	6	5	9	15	No	MV	No
11	2022-09-19	5	4	7	8	10	4	MV/HFNC	Yes
12	2022-04-01	9	5	6	6	9	5	MV/HFNC	No
13	2022-03-31	8	7	8	7	11	5	MV	No
14	2022-04-12	5	8	5	8	12	No	MV	No
15	2022-04-08	7	15	6	10	15	5	MV	Yes
16	2022-03-15	8	5	5	5	10	No	MV	No

MV, mechanical ventilation; HFNC, high-flow nasal cannula oxygen therapy; CRRT, continuous renal replacement therapy.

## Discussion

In this study, we conducted a retrospective review of 16 patients diagnosed with *Chlamydia psittaci* infection by the application of mNGS, manifesting as severe pneumonia with ARDS. Human infection of psittacosis was commonly known as inhalation of feather, feces, or corpses of birds or poultry, contact with their excreta, activities that involve cooking or pruning, and cleaning of contaminated cages (20, 21). Transmission from human to human by psittacosis is considered rare due to a few reports of psittacosis outbreaks in humans (22). Studies have reported that 90% of patients infected with *Chlamydia psittaci* had bird or poultry contact history with seropositivity rates of 13.3%, 38.9%, and 31.1% in chickens, ducks, and pigeons sold in Northwest China, respectively (23, 24). In the study, all patients had a direct or indirect exposure to parrots, pigeons, chickens, or ducks, which was considered the main risk factor for psittacosis. Additionally, 8 out of the 16 patients with underlying diseases had moderate to severe ARDS, suggesting that *Chlamydia psittaci* could infect human subjects as a critical illness condition.

The lungs are the most common sites of *Chlamydia psittaci* infection after entry through contaminated aerosols. Probably owing to the atypical clinical manifestations and relatively low awareness by physicians, the diagnosis of *Chlamydia psittaci* pneumonia is challenging and tends to be overlooked with the lack of routine diagnostic methods. Patients are usually treated for common CAP when the lack or omission of contact history collected in medical history. The misdiagnosis of *Chlamydia psittaci* pneumonia may also be increased due to the indistinguishable symptoms,familial aggregation and lack of medical resources under COVID-19. Furthermore, measurement of *Chlamydia psittaci* is not often available in the routine test. Thus, patients are mainly administered empirical antibiotics, which potentially leads to misdiagnosis and underestimation of the accurate incidence of *Chlamydia psittaci* pneumonia (25). The typical symptoms include fever, chill, cough, and myalgia (26, 27). In the study, almost all patients had fever, dyspnea, and cough. All patients had hypoxemia, resulting in ARDS during the process. Some patients had headache at the onset of disease. Headache is rarely reported in *Chlamydia psittaci* pneumonia. It has been reported that the cases infected with *Chlamydia psittaci* to the central nervous system had poor outcomes with the unclear mechanisms (28). Headache should draw an attention to the possible lung infection if it cannot be explained by neurological disease. The major manifestations of chest CT are consolidation, ground glass shadow, lobular distribution, and pleural effusion mainly located in the lower lobes of the lungs (29, 30). In the study, there was one patient coexisted with lobe lesions and pleural effusion, indicating the gradually development from the lung to pleural cavity. Furthermore, *Chlamydia psittaci* is associated with several extrapulmonary manifestations, including myocarditis, endocarditis, arthritis, hepatitis, encephalitis, and ocular adnexal lymphoma (31). Therefore, difficulty in differentiating *Chlamydia psittaci* pneumonia from other pathogens causing CAP based on the absence of characteristic clinical manifestations and images can lead to misdiagnosis and delay in treatment. This will indicate more information of *Chlamydia psittaci* pneumonia for us.

In the current study, the laboratory data of patients generally showed normal leucocytes, increased neutrophils, CRP, and PCT. *Chlamydia psittaci* has been reported to be more pathogenic than other Chlamydiales species, causing more severe inflammatory reactions (27). In another case, a significantly lower level of leucocytes was also reported (32). All patients had decreased lymphocyte counts, indicating immune dysfunction associated with psittacosis infection. Lymphocytopenia is a common symptom in patients with severe community-acquired infections by the destruction of the cytoplasmic components and apoptosis of lymphocytes with some atypical pathogens (33). However, the reason of lymphocytopenia caused by *Chlamydia psittaci* still needs further investigation. Most patients had an elevated level of CK-MB, ALT, and AST, suggesting multiple organ dysfunction such as myocardial damage and hepatic dysfunction by *Chlamydia psittaci* infection as previously reported (25). CK is considered a high-risk factor for severe *Chlamydia psittaci* pneumonia that is not rare in clinics (34). In addition, changes in liver enzymes are attributed to the entrance of the reticular endothelial cells of the liver by *Chlamydia psittaci*. When patients with fever, increased liver enzyme, or nervous system with an age of <60 years, the diagnosis of *Chlamydia psittaci* should be considered to avoid ARDS or septic shock at a later stage (35). Healthcare providers should be on high alert when patients present with these conditions in order to avoid severe sequelae. Furthermore, we also found a relatively high incidence of PE with an increased level of plasma D-dimer in severe *Chlamydia psittaci* pneumonia that had not been reported before. The mechanism of PE by psittacosis infection still remained unclear. Patients with PE received anticoagulant therapy due to the definite aggravation of hypoxemia in the condition of ARDS.

Laboratory testing for *Chlamydia psittaci* has drawn attention with the lack of ideal method tools. Routine sputum culture usually takes 5–7 days with a low sensitive rate of *Chlamydia psittaci* and hazardous for personnel in a P3 containment laboratory (36). The serological assay has a cross-reaction with other Chlamydia species and is appropriate for retrospective diagnosis and epidemiological investigation in acute and convalescent stages (37). PCR can help identify the pathogen in a more rapid, sensitive, and specific way during the acute phases of the infection (9). However, PCR for *Chlamydia psittaci* is unavailable in most hospitals in China. The advantage of mNGS is its quick and accurate detection of lower respiratory tract infections in a wide range of pathogens, including atypical bacterial pathogens, viruses, and fungi. In our hospital, the results of mNGS are available within 24–48 h, which is beneficial and important for accurate diagnosis and targeted treatment as early as possible when treating patients with severe *Chlamydia psittaci* pneumonia. In the current study, all patients were diagnosed and adjusted treatment through mNGS after no improvement with empirical antibiotic therapy. It is noteworthy that the diagnosis of severe CAP caused by *Chlamydia psittaci* with the application of mNGS presents a valuable method in timely therapy.

The sensitivity of *Chlamydia psittaci* is relatively low because of its intracellular bacterium and the small number of body fluids such as blood, sputum, and BALF by mNGS detection. Therefore, even with small amounts of DNA sequence, *Chlamydia psittaci* should be considered as the causative pathogen for pneumonia in the study. In addition, some patients had other pathogens detected by mNGS. Patients with underlying diseases or immunocompromised condition, bacteria colonization, or workflow quality may affect the interpretation of mNGS results. It is required the ability of clinicians to be strict enough for interpretation of mNGS results.

There is a higher rate of incidence of severe CAP caused by *Chlamydia psittaci*. The mortality rate was 15%–20% before the targeted antibiotics were used (38). Antibiotics, including tetracycline, macrolide, and quinolones, are recommended for *Chlamydia psittaci* pneumonia treatment (39). The duration of treatment should continue for at least 10–14 days to prevent relapse (40). Quinolones have been reported to be less effective compared with tetracyclines and macrolides in psittaci pneumonia (41). In our study, patients who received moxifloxacin/ levofloxacin treatment initially had poor response, covering the targeted pathogen prior to diagnosis, especially in severe conditions. The possible reason may be related to the low intracellular activity of quinolones in *Chlamydia psittaci* and the insensitivity of *Chlamydia psittaci* response to quinolones. Tetracycline is the first-line treatment recommended for *Chlamydia psittaci* pneumonia (42). Omadacycline, a third-generation tetracycline, has a structure of an alkylaminomethyl group that replaces the glycylamido group at the C-9 position of the D-ring derived from minocycline that is helpful for bacterial resistance (16, 43). Unlike other tetracyclines, omadacycline inhibits bacterial protein synthesis by only binding to the 30S ribosomal subunit and not binding to the 50S ribosomal subunit. As we know, doxycycline and minocycline are prone to drug-induced organic injury with liver metabolism (44). On the contrary, omadacycline has a higher concentration in the lung than the plasma and is eliminated mainly by feces which is not required for dose adjustment in the elderly and patients with hepatic and renal impairment (45). In the study, most patients showed good efficacy and recovery after the administration of omadacycline. Severe *Chlamydia psittaci* pneumonia can lead to multiple organ failure. All patients had ARDS with septic shock, which need MV and organ support therapy. In the present study, patients with PaO_2_/FIO_2_ ratio <150 mmHg received prone position. Four patients with acute renal injury were treated with CRRT. Additionally, patients with septic shock and MV were susceptible to secondary infections, as illustrated by the patient who died from multiple drug-resistant bacterial infections. To the best of our knowledge, this is the first report of omadacycline for the treatment of severe *Chlamydia psittaci* pneumonia with ARDS in China.

This study had some limitations: (1) the number of the critically ill patients was relatively small and was insufficient to investigate the relevant data of psittacosis pneumonia; (2) there was a lack of verification with serologic tests in the current study because the requirements were unavailable in our hospital; (3) the retrospective nature of the study provided limited evidence of the therapeutic effect of omacycline. A prospective study with larger sample sizes is ongoing to confirm our findings.

## Conclusion

Severe CAP caused by *Chlamydia psittaci* could lead to ARDS or sepsis or even multiple organ failure. mNGS is a valuable method for accurate diagnosis and targeted antibiotic treatment. mNGS can save time to diagnose and identify pathogens, especially important for those with poor response to empirical antibiotics. This study showed that omadacycline may be considered as a new option for the treatment of *Chlamydia psittaci* pneumonia with ARDS.

## Data availability statement

The data presented in the study are deposited in the China National GeneBank DataBase (https://db.cngb.org/) accession number CNP0004281.

## Ethics statement

The studies involving humans were approved by the Second Affiliated Hospital of Chongqing Medical University. The studies were conducted in accordance with the local legislation and institutional requirements. Written informed consent for participation was not required from the participants or the participants’ legal guardians/next of kin because given the retrospective nature of the study. Written informed consent was obtained from the individual(s) for the publication of any potentially identifiable images or data included in this article.

## Author contributions

WD proposed the idea of the study, participated in the design of the study, and revised the manuscript. D-XW drafted the manuscript. L-XX collected and analyzed clinical data. X-YD performed data interpretation. All authors contributed to the article and approved the submitted version.

## References

[1] Pannekoek Y Visser C Duim B Heddema ER. *Chlamydophila psittaci* infections in the Netherlands. Drugs Today. (2009) 45:151–7. PMID: 20011708

[2] Hogerwerf L Roof I de Jong MJK Dijkstra F van der Hoek W. Animal sources for zoonotic transmission of psittacosis: a systematic review. BMC Infect Dis. (2020) 20:192. doi: 10.1186/s12879-020-4918-y, PMID: 32131753 PMC7057575

[3] Chen X Cao K Wei Y Qian Y Liang J Dong D. Metagenomic next-generation sequencing in the diagnosis of severe pneumonias caused by *Chlamydia psittaci*. Infection. (2020) 48:535–42. doi: 10.1007/s15010-020-01429-0, PMID: 32314307 PMC7223968

[4] Qu J Zhang J Chen Y Huang Y Xie Y Zhou M. Aetiology of severe community acquired pneumonia in adults identified by combined detection methods: a multi-centre prospective study in China. Emerg Microbes Infect. (2022) 11:556–66. doi: 10.1080/22221751.2022.2035194, PMID: 35081880 PMC8843176

[5] Balsamo G Maxted AM Midla JW Murphy JM Wohrle R Edling TM. Compendium of measures to control *Chlamydia psittaci* infection among humans (psittacosis) and pet birds (avian chlamydiosis). J Avian Med Surg. (2017) 31:262–82. doi: 10.1647/217-265, PMID: 28891690

[6] Wang L Shi Z Chen W Du X Zhan L. Extracorporeal membrane oxygenation in severe acute respiratory distress syndrome caused by *Chlamydia psittaci*: a case report and review of the literature. Front Med. (2021) 8:731047. doi: 10.3389/fmed.2021.731047, PMID: 34722571 PMC8554049

[7] Branley JM Weston KM England J Dwyer DE Sorrell TC. Clinical features of endemic community-acquired psittacosis. New Microbes New Infect. (2014) 2:7–12. doi: 10.1002/2052-2975.29, PMID: 25356332 PMC4184616

[8] Rybarczyk J Versteele C Lernout T Vanrompay D. Human psittacosis: a review with emphasis on surveillance in Belgium. Acta Clin Belg. (2020) 75:42–8. doi: 10.1080/17843286.2019.1590889, PMID: 30882289

[9] Nieuwenhuizen AA Dijkstra F Notermans DW van der Hoek W. Laboratory methods for case finding in human psittacosis outbreaks: a systematic review. BMC Infect Dis. (2018) 18:442. doi: 10.1186/s12879-018-3317-0 30165831 PMC6118005

[10] Smith KA Bradley KK Stobierski MG Tengelsen LA. National Association of State Public Health Veterinarians Psittacosis Compendium Committee. Compendium of measures to control *Chlamydophila psittaci* (formerly *Chlamydia psittaci*) infection among humans (psittacosis) and pet birds, 2005. J Am Vet Med Assoc. (2005) 226:532–9. doi: 10.2460/javma.2005.226.532 15742693

[11] Ménard A Clerc M Subtil A Mégraud F Bébéar C de Barbeyrac B. Development of a real-time PCR for the detection of *Chlamydia psittaci*. J Med Microbiol. (2006) 55:471–3. doi: 10.1099/jmm.0.46335-0 16533998

[12] de Gier B Hogerwerf L Dijkstra F van der Hoek W. Disease burden of psittacosis in the Netherlands. Epidemiol Infect. (2018) 146:303–5. doi: 10.1017/S0950268817003065, PMID: 29361998 PMC9134569

[13] Schlaberg R Chiu CY Miller S Procop GW Weinstock G Professional practice committee and committee on laboratory practices of the American Society for Microbiology; Microbiology Resource Committee of the College of American Pathologists. Validation of metagenomic next-generation sequencing tests for universal pathogen detection. Arch Pathol Lab Med. (2017) 141:776–6. doi: 10.5858/arpa.2016-0539-RA 28169558

[14] Zhou H Larkin PMK Zhao D Ma Q Yao Y Wu X. Clinical impact of metagenomic next-generation sequencing of bronchoalveolar lavage in the diagnosis and management of pneumonia: a multicenter prospective observational study. J Mol Diagn. (2021) 23:1259–68. doi: 10.1016/j.jmoldx.2021.06.007, PMID: 34197923

[15] Langelier C Kalantar KL Moazed F Wilson MR Crawford ED Deiss T. Integrating host response and unbiased microbe detection for lower respiratory tract infection diagnosis in critically ill adults. Proc Natl Acad Sci U S A. (2018) 115:E12353–62. doi: 10.1073/pnas.1809700115, PMID: 30482864 PMC6310811

[16] Zhanel GG Esquivel J Zelenitsky S Lawrence CK Adam HJ Golden A. Omadacycline: a novel oral and intravenous aminomethylcycline antibiotic agent. Drugs. (2020) 80:285–313. doi: 10.1007/s40265-020-01257-4, PMID: 31970713

[17] Metlay JP Waterer GW Long AC Anzueto A Brozek J Crothers K. Diagnosis and treatment of adults with community-acquired pneumonia. An official clinical practice guideline of the American Thoracic Society and Infectious Diseases Society of America. Am J Respir Crit Care Med. (2019) 200:e45–67. doi: 10.1164/rccm.201908-1581ST 31573350 PMC6812437

[18] ARDS Definition Task Force Ranieri VM Rubenfeld GD Thompson BT Ferguson ND Caldwell E. Acute respiratory distress syndrome: the Berlin definition. JAMA. (2012) 307:2526–33. doi: 10.1001/jama.2012.5669 22797452

[19] Papazian L Aubron C Brochard L Chiche JD Combes A Dreyfuss D. Formal guidelines: management of acute respiratory distress syndrome. Ann Intensive Care. (2019) 9:69. doi: 10.1186/s13613-019-0540-9, PMID: 31197492 PMC6565761

[20] Fraeyman A Boel A Van Vaerenbergh K De Beenhouwer H. Atypical pneumonia due to *Chlamydophila psittaci*: 3 case reports and review of literature. Acta Clin Belg. (2010) 65:192–6. doi: 10.1179/acb.2010.040, PMID: 20669788

[21] Lagae S Kalmar I Laroucau K Vorimore F Vanrompay D. Emerging *Chlamydia psittaci* infections in chickens and examination of transmission to humans. J Med Microbiol. (2014) 63:399–407. doi: 10.1099/jmm.0.064675-0, PMID: 24324029

[22] Li N Li S Tan W Wang H Xu H Wang D. Metagenomic next-generation sequencing in the family outbreak of psittacosis: the first reported family outbreak of psittacosis in China under COVID-19. Emerg Microbes Infect. (2021) 10:1418–28. doi: 10.1080/22221751.2021, PMID: 34176434 PMC8284143

[23] Hulin V Bernard P Vorimore F Aaziz R Cléva D Robineau J. Assessment of *Chlamydia psittaci* shedding and environmental contamination as potential sources of worker exposure throughout the mule duck breeding process. Appl Environ Microbiol. (2015) 82:1504–18. doi: 10.1128/AEM.03179-15, PMID: 26712548 PMC4771335

[24] Cong W Huang SY Zhang XY Zhou DH Xu MJ Zhao Q. Seroprevalence of *Chlamydia psittaci* infection in marketsold adult chickens, ducks and pigeons in north-western China. J Med Microbiol. (2013) 62:1211–4. doi: 10.1099/jmm.0.059287-0 23699067

[25] Rane V Khailin K Williams J Francis M Kotsanas D Korman TM. Underdiagnosis of *Chlamydia trachomatis* and *Chlamydia psittaci* revealed by introduction of respiratory multiplex PCR assay with Chlamydiaceae family primers. Diagn Microbiol Infect Dis. (2018) 90:163–6. doi: 10.1016/j.diagmicrobio.2017.11.013, PMID: 29258707

[26] Hogerwerf L de Gier B Baan B van der Hoek W. *Chlamydia psittaci* (psittacosis) as a cause of community-acquired pneumonia: a systematic review and meta-analysis. Epidemiol Infect. (2017) 145:3096–105. doi: 10.1017/S0950268817002060, PMID: 28946931 PMC9148753

[27] Knittler MR Sachse K. *Chlamydia psittaci*: update on an underestimated zoonotic agent. Pathog Dis. (2015) 73:1–15. doi: 10.1093/femspd/ftu007, PMID: 25853998

[28] Walder G Schönherr H Hotzel H Speth C Oehme A Dierich MP. Presence of *Chlamydophila psittaci* DNA in the central nervous system of a patient with status epilepticus. Scand J Infect Dis. (2003) 35:71–3. doi: 10.1080/0036554021000026984, PMID: 12685890

[29] Longbottom D Coulter LJ. Animal chlamydioses and zoonotic implications. J Comp Pathol. (2003) 128:217–44. doi: 10.1053/jcpa.2002.0629, PMID: 12834606

[30] Zucca F Bertoni F. Chlamydia or not Chlamydia, that is the question: which is the microorganism associated with MALT lymphomas of the ocular adnexa? J Natl Cancer Inst. (2006) 98:1348–9. doi: 10.1093/jnci/djj406 17018775

[31] Schlossberg D. Psittacosis (due to *Chlamydia psittaci*) In: Bennett JE Dolin R Blaser MJ, editors. Mandell, Douglas, and Bennett’s principles and practice of infectious diseases. 8th ed. Philadelphia, PA: Elsevier/Saunders (2015). 2171–3.

[32] Vande Weygaerde Y Versteele C Thijs E De Spiegeleer A Boelens J Vanrompay D. An unusual presentation of a case of human psittacosis. Respir Med Case Rep. (2018) 23:138–42. doi: 10.1016/j.rmcr.2018.01.010, PMID: 29719801 PMC5926501

[33] Gu J Gong E Zhang B Zheng J Gao Z Zhong Y. Multiple organ infection and the pathogenesis of SARS. J Exp Med. (2005) 202:415–24. doi: 10.1084/jem.20050828, PMID: 16043521 PMC2213088

[34] Yang F Li J Qi B Zou L Shi Z Lei Y. Clinical symptoms and outcomes of severe pneumonia caused by *Chlamydia psittaci* in southwest China. Front Cell Infect Microbiol. (2022) 11:727594. doi: 10.3389/fcimb.2021.727594, PMID: 35071027 PMC8770948

[35] Raeven VM Spoorenberg SM Boersma WG van de Garde EM Cannegieter SC Voorn GP. Atypical aetiology in patients hospitalised with community-acquired pneumonia is associated with age, gender and season; a data-analysis on four Dutch cohorts. BMC Infect Dis. (2016) 16:299. doi: 10.1186/s12879-016-1641-9 27317257 PMC4912822

[36] Wilson MR Sample HA Zorn KC Arevalo S Yu G Neuhaus J. Clinical metagenomic sequencing for diagnosis of meningitis and encephalitis. N Engl J Med. (2019) 380:2327–40. doi: 10.1056/NEJMoa1803396, PMID: 31189036 PMC6764751

[37] Spoorenberg SM Bos WJ van Hannen EJ Dijkstra F Heddema ER van Velzen-Blad H. *Chlamydia psittaci*: a relevant cause of community-acquired pneumonia in two Dutch hospitals. Neth J Med. (2016) 74:75–81. PMID: 26951352

[38] Wu HH Feng LF Fang SY. Application of metagenomic next-generation sequencing in the diagnosis of severe pneumonia caused by *Chlamydia psittaci*. BMC Pulm Med. (2021) 21:300. doi: 10.1186/s12890-021-01673-6, PMID: 34556069 PMC8461849

[39] Cillóniz C Torres A Niederman M van der Eerden M Chalmers J Welte T. Community-acquired pneumonia related to intracellular pathogens. Intensive Care Med. (2016) 42:1374–86. doi: 10.1007/s00134-016-4394-4, PMID: 27276986

[40] Cao B Huang Y She DY Cheng QJ Fan H Tian XL. Diagnosis and treatment of community-acquired pneumonia in adults: 2016 clinical practice guidelines by the Chinese Thoracic Society, Chinese Medical Association. Clin Respir J. (2018) 12:1320–60. doi: 10.1111/crj.12674, PMID: 28756639 PMC7162259

[41] de Boeck C Dehollogne C Dumont A Spierenburg M Heijne M Gyssens I. Managing a cluster outbreak of psittacosis in Belgium linked to a pet shop visit in the Netherlands. Epidemiol Infect. (2016) 144:1710–6. doi: 10.1017/S0950268815003106, PMID: 26669637 PMC9150705

[42] Mandell LA Wunderink RG Anzueto A Bartlett JG Campbell GD Dean NC. Infectious Diseases Society of America/American Thoracic Society consensus guidelines on the management of community-acquired pneumonia in adults. Clin Infect Dis. (2007) 44:S27–72. doi: 10.1086/511159, PMID: 17278083 PMC7107997

[43] Sakoulas G Nowak M Geriak M. Omadacycline in treating community-based infections: a review and expert perspective. Expert Rev Anti-Infect Ther. (2023) 21:255–65. doi: 10.1080/14787210.2023.2174100, PMID: 36718489

[44] Wei C Liu Y Jiang A Wu B. A pharmacovigilance study of the association between tetracyclines and hepatotoxicity based on Food and Drug Administration adverse event reporting system data. Int J Clin Pharm. (2022) 44:709–16. doi: 10.1007/s11096-022-01397-5, PMID: 35364753

[45] Rodvold KA Burgos RM Tan X Pai MP. Omadacycline: a review of the clinical pharmacokinetics and pharmacodynamics. Clin Pharmacokinet. (2020) 59:409–25. doi: 10.1007/s40262-019-00843-4, PMID: 31773505

